# HSP27 Inhibitory Activity against Caspase-3 Cleavage and Activation by Caspase-9 Is Enhanced by Chaperone O-GlcNAc Modification *in Vitro*

**DOI:** 10.1021/acschembio.3c00270

**Published:** 2023-07-14

**Authors:** Binyou Wang, Stuart P. Moon, Giuliano Cutolo, Afraah Javed, Benjamin S. Ahn, Andrew H. Ryu, Matthew R. Pratt

**Affiliations:** ^†^Department of Chemistry and ^‡^Biological Sciences, University of Southern California, Los Angeles, California 90089, United States

## Abstract

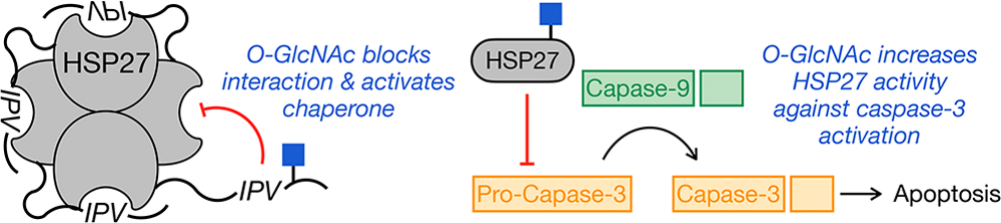

One of the O-GlcNAc modifications is the protection of cells against a variety of stressors that result in cell death. Previous experiments have focused on the overall ability of O-GlcNAc to prevent protein aggregation under stress as well as its ability to affect stress-response signaling pathways. Less attention has been paid to the potential role for O-GlcNAc in the direct inhibition of a major cell-death pathway, apoptosis. Apoptosis involves the sequential activation of caspase proteases, including the transfer of cell-stress information from initiator caspase-9 to effector caspase-3. Cells have multiple mechanisms to slow the apoptotic cascade, including heat shock protein HSP27, which can directly inhibit the activation of caspase-3 by caspase-9. We have previously shown that O-GlcNAc modification increases the chaperone activity of HSP27 against amyloid aggregation, raising the question as to whether this modification may play important roles in other facets of HSP27 biology. Here, we use protein chemistry to generate different versions of O-GlcNAc modified HSP27 and demonstrate that the modification enhances this antiapoptotic function of the chaperone, at least in an *in vitro* context. These results provide additional molecular insight into how O-GlcNAc functions as a mediator of cellular stress with important implications for human diseases like cancer and neurodegeneration.

## Introduction

**Figure 1:**
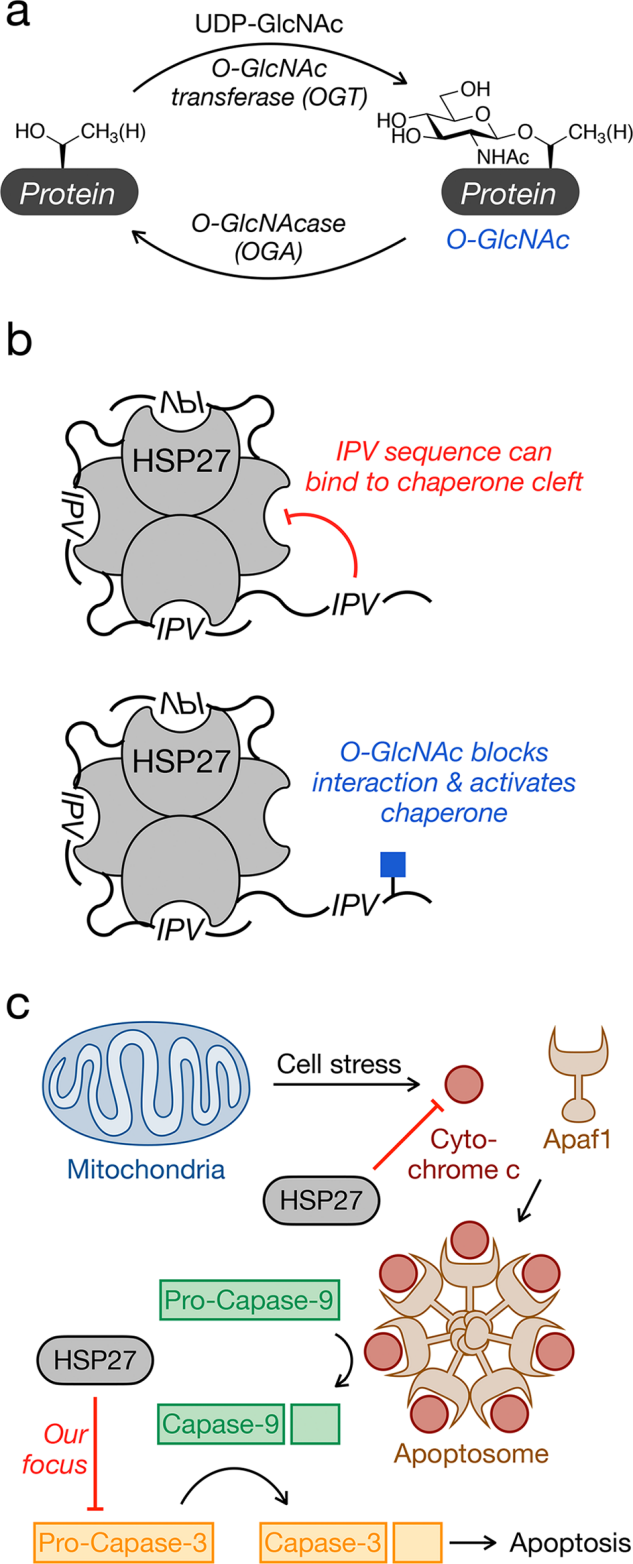
O-GlcNAc, HSP27, and apoptosis. a) O-GlcNAc is the dynamic addition of *N*-acetylglucosamine to intracellular proteins. b) HSP27 functions as an oligomer, where an IPV sequence in the *C*-terminal tail can bind to the chaperone cleft and regulate its activity. We found that the presence of an O-GlcNAc near the IPV blocks this interaction and activates the chaperone. c) The intrinsic pathway of apoptosis begins with the release of cytochrome c from the mitochondria where it can form the apoptosome with another protein, Apaf1. The apoptosome recruits caspase-9 resulting in its cleavage and activation. Caspase-9 then cleaves downstream caspases, including caspase-3, that go on to proteolyze hundreds of substrates, resulting in cell death.

O-GlcNAc modification (Figure 1a), an intracellular form of glycosylation, plays critical roles in metazoan survival and cell death.^1−3^ This post-translational modification (PTM) is the addition of the monosaccharide *N*-acetyl-glucosamine to serine and threonine side-chains of intracellular proteins. The levels and dynamics of O-GlcNAc are controlled by several factors, including the availability of nutrients, cellular stress, and the expression of O-GlcNAc transferase (OGT) and O-GlcNAcase (OGA).^4^ These two enzymes add and remove O-GlcNAc respectively and enable the dynamic cycling of this modification in response to various cellular stimuli. O-GlcNAc is intimately linked with cell survival. Knockout of OGT in neurons or T-cells results in programmed cell death or apoptosis.^5^ The overall levels of O-GlcNAc are higher in almost every type of cancer and tumor that has been tested.^6−8^ Reduction of the level of O-GlcNAcylation in cancer cells using either RNAi or small molecule inhibitors reduces tumorigenesis and metastasis and causes apoptosis. For example, we demonstrated that inhibiting the biosynthesis of UDP-GlcNAc, the substrate for OGT, increases the sensitivity of breast and lung cancer cells to apoptosis induced by oxidative stress.^9^ O-GlcNAc levels are also rapidly increased by a variety of stressors (heat, osmotic pressure, UV light, hypoxia, etc.), as well as heart attack and ischemia.^10,11^ Preventing this increase diminishes the resistance of cells to stress and induces more apoptosis. Despite these strong phenotypic associations, the direct molecular mechanisms by which O-GlcNAc inhibits cell death by O-GlcNAc is still being uncovered.

Similar to the case for O-GlcNAc, heat shock proteins are upregulated in cancer and under cellular stress. The small heat shock proteins (sHSPs) are ATP-independent chaperones that typically bind to un(mis)folded proteins and physically prevent their aggregation.^12,13^ A major sHSP is HSP27, which is broadly expressed in mammalian tissues. HSP27 forms oligomers that can intercept and “shield” unfolded proteins or protein segments, promoting the formation of highly soluble complexes.^14^ HSP27 is composed of three domains: an unfolded N-terminus, a central α-Crystallin domain (ACD), and a C-terminal tail containing an IPV sequence (Figure 1b).^15,16^ The N- and C-termini are important for the formation of HSP27 oligomers, and a β-cleft in the ACD is the major site of substrate–protein binding. The IPV sequence in the C-terminal tail is also involved in the regulation of the HSP27 activity. Briefly, this IPV can compete with substrate for binding to the ACD cleft.^17−20^ HSP27 has been identified as an O-GlcNAc modified protein, with the O-GlcNAc modification sites found near the IPV.^21−23^ This led us to previously hypothesize that the O-GlcNAc would inhibit the IPV-ACD interaction and activate the chaperone. To test this hypothesis, we used synthetic protein chemistry to prepare HSP27 bearing site-specific O-GlcNAc modifications and found that the glycosylation did indeed disrupt the IPV-ACD association and make HSP27 a better chaperone against amyloid aggregation.^24^ While this result has important implications in neurodegenerative diseases, HSP27 has other functions, including the inhibition of apoptosis, raising the possibility that the O-GlcNAc pathway may affect this pathway too.

The caspase family of cysteine proteases carries out apoptosis. The caspases are expressed as zymogens that undergo cleavage and activation upon various apoptotic inputs. Apoptosis can be broadly separated into two major pathways: intrinsic and extrinsic apoptosis. Both pathways begin with the oligomerization of an initiator caspase, resulting in its autoactivation. The initiator caspase then cleaves and activates downstream executioner caspases that then move to cleave hundreds of proteins that result in programmed cell death. The intrinsic apoptotic pathway is highlighted in Figure 1c and largely results from intracellular signals. Briefly, cytochrome c is released by mitochondria, where it can form a complex with another protein Apaf1 and recruit caspase-9. The oligomerization of caspase-9 in this apoptosome results in its self-cleavage, and the activated protease then cleaves the executioner caspases, particularly caspase-3. As noted above, HSP27 has been shown to inhibit the intrinsic apoptotic pathway by at least two mechanisms (Figure 1c). HSP27 can bind to cytochrome-c to prevent the formation of the apoptosome^25^ and interact with caspase-3 to prevent its activation by caspase-9.^26^ More specifically, HSP27 binds to the unstructured N-terminus (pro-domain) of caspase-3 and thus prevents binding of caspase-9, cleavage/activation of caspase-3, and apoptosis.^26^

Here, we used synthetic proteins to discover that O-GlcNAc increases the antiapoptotic activity of HSP27 *in vitro*. We first synthesized HSP27 bearing an O-GlcNAc at the likely major site of modification, threonine 184, as it the only site that has been identified in every proteomics data set.^21−23^ As in our published work, we initially removed an endogenous cysteine at residue 137 for ease of synthesis yielding a protein we term HSP27(gT184) (C137A), and we and others have shown that this mutation does not dramatically affect the antiaggregation activity of HSP27. Using assays for direct activation of caspase-3 by caspase-9, we then demonstrated that HSP27 O-GlcNAc modification slows this activation cascade compared to the unmodified chaperone. Notably, in the study highlighted above, the authors found that overexpressed HSP27 required C137 to bind cytochrome c and inhibit the formation of the apoptosome,^25^ and C137 has been shown to regulate HSP27 dimer to monomer transitions as a potential sensor of oxidative conditions.^27^ While we are unable to directly compare the overexpression experiment using synthetic proteins, we wondered whether C137 may also be important in the case of direct caspase-3 activation by caspase-9. Therefore, we next invested significant synthetic effort into the synthesis of O-GlcNAc modified HSP27(gT184), containing the native cysteine. With this protein in hand, we confirmed the importance of C137, as well as the enhancement of HSP27 antiapoptotic activity by O-GlcNAc. These results build upon our data showing that O-GlcNAc modification of HSP27 by O-GlcNAc likely plays an important role in protein aggregation and extends this biochemistry to apoptosis. Given that the level of concentration of O-GlcNAc is elevated in cancer, we believe this may have important mechanistic implications for how the modification promotes cancer cell survival and growth.

## Results and Discussion

**Figure 2:**
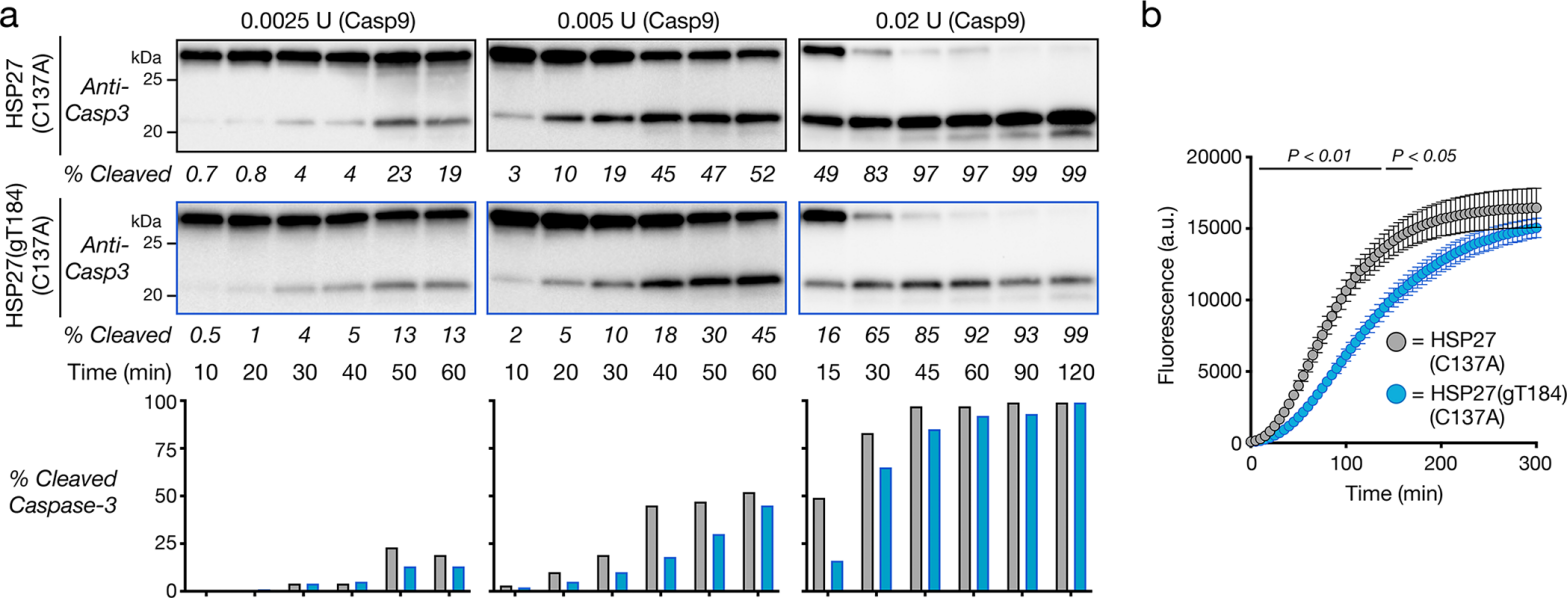
O-GlcNAc on HSP27 increases the inhibition of caspase-3 cleavage by caspase-9. a) HSP27 or its O-GlcNAc-modified counterpart, HSP27(gT184), was incubated with pro-caspase-3 before addition of the indicated amounts of caspase-9 and the levels of caspase-3 cleavage were measured by Western blotting. b) A similar experiment to “a” was performed and caspase-3 activity was measured using a fluorescent substrate. Results are mean ± SEM of experimental replicates (*n* = 6). Statistical significance was determined using two-tailed student’s *t* tests at each time point.

To test whether O-GlcNAc increases the ability of HSP27 to block caspase-3 activation by caspase-9, we first recombinantly expressed the unmodified HSP27(C137A) and synthesized the O-GlcNAc-modified species according to our published method.^24^ With these proteins in hand, we individually incubated them with recombinant pro-caspase-3 at a 3:1 ratio of chaperone to caspase. We chose this ratio, as it was enough chaperone to notably slow caspase-3 activation without blocking it completely. After 30 min, we then added recombinant, active caspase-9 and analyzed the cleavage, and thus activation, of caspase-3 by Western blotting (Figure 2a). Notably, we performed this reaction with three different amounts of caspase-9 to capture the effect of O-GlcNAc under different kinetic parameters. In all three conditions, we consistently observed less caspase-3 cleavage in the presence of HSP27(gT184) (C137A) compared with its unmodified counterpart. To confirm these results, we also used a fluorescence assay that reads caspase-3 catalytic activity (Figure 2b). Again, we detected a statistically significant delay in the induction of caspase-3 activity in the reactions containing the O-GlcNAc modified HSP27.

**Figure 3:**
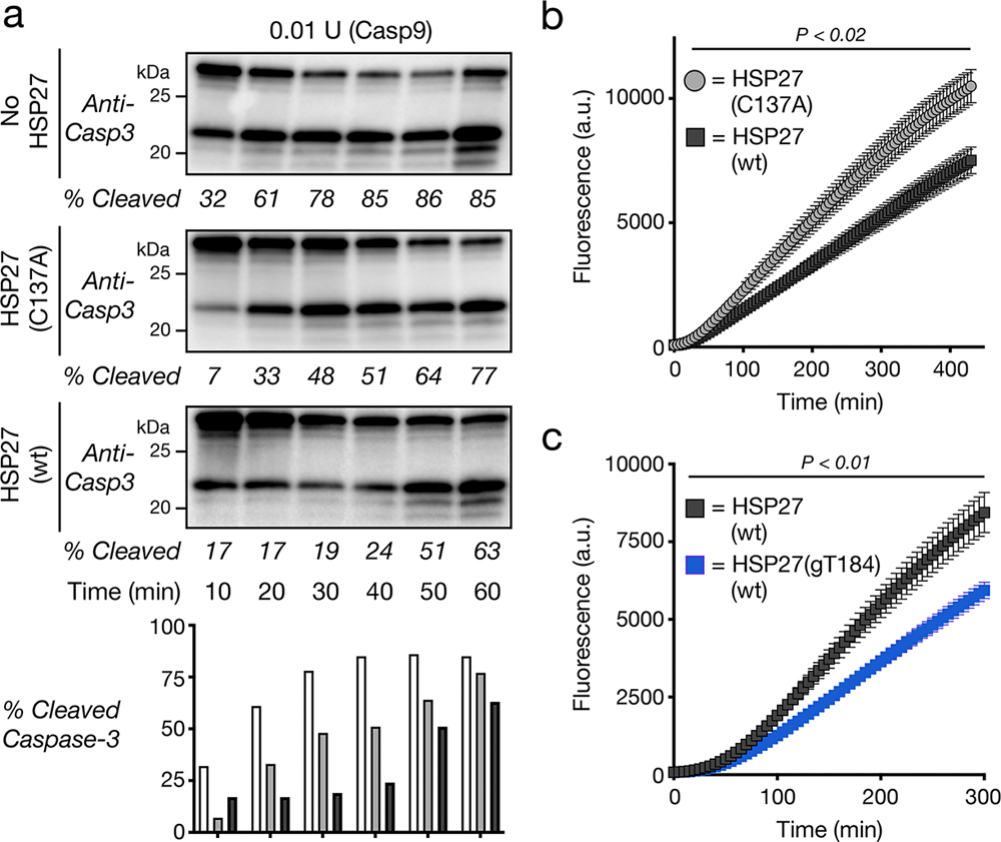
Both C137 and the O-GlcNAc modification at T184 increase the inhibitory activity of HSP27 a) HSP27(C137A) or its wild-type counterpart, HSP27(wt), were incubated with pro-caspase-3 before addition of the indicated amounts of caspase-9 and the levels of caspase-3 cleavage were measured by Western blotting. b) A similar experiment to “a” was performed in triplicate and caspase-3 activity was measured using a fluorescent substrate. Results are mean ± SEM of experimental replicates (*n* = 6). Statistical significance was determined using two-tailed student’s *t* tests at each time point. c) O-GlcNAc of HSP27(wt) further increases the inhibition of caspase-3 activation. The conditions in Figure 2b were repeated with wild-type proteins, and caspase-3 activity was measured using a fluorescent substrate. Results are mean ± SEM of experimental replicates (*n* = 4). Statistical significance was determined using two-tailed student’s *t* tests at each time point.

**Figure 4:**
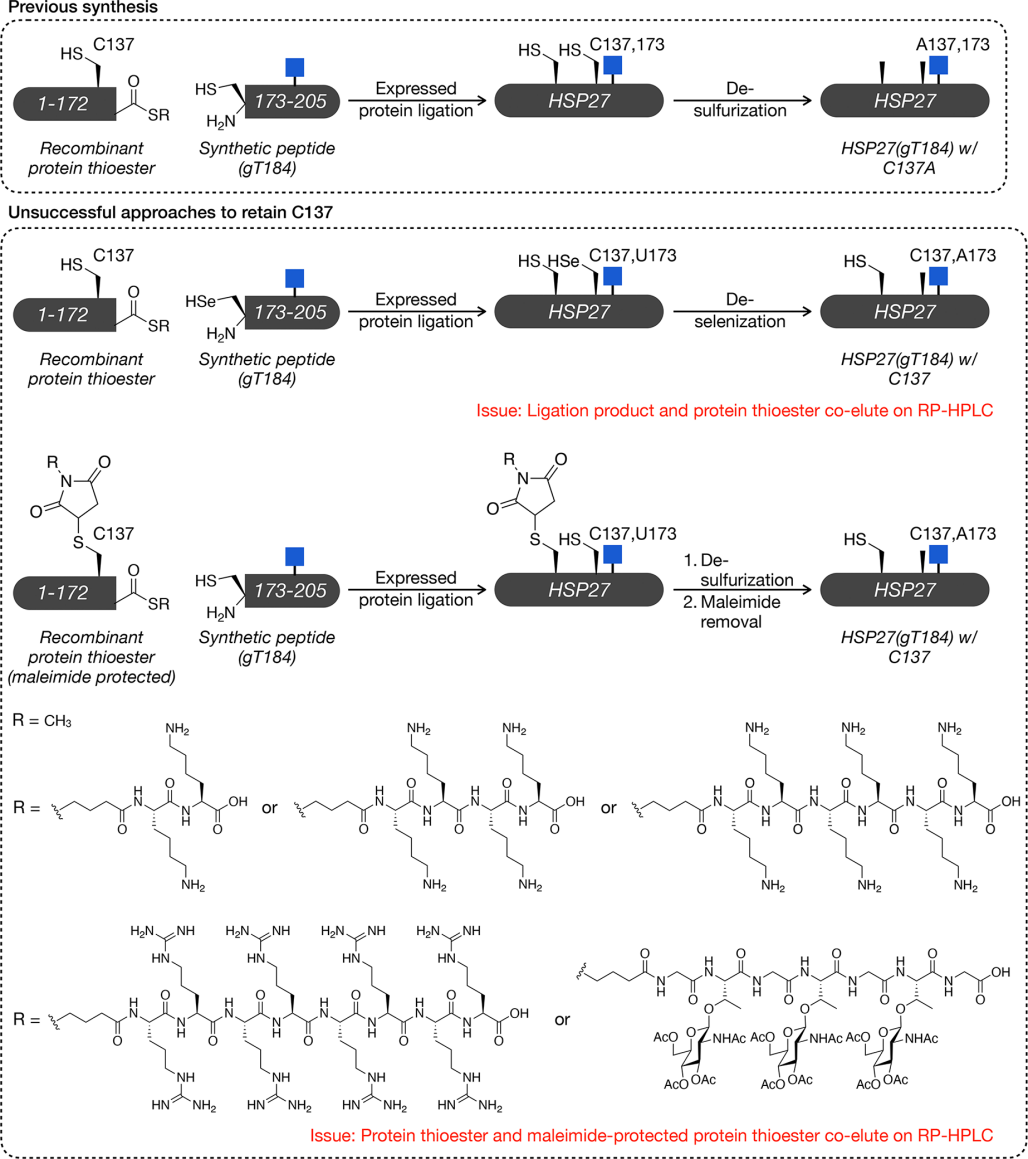
Synthesis of O-GlcNAc modified HSP27 Our previously published synthesis retained both C137 and the cystine required for ligation at residue 173 before desulfurization of both cysteines to give HSP27(gT184) (C137A). Here, we attempted several approaches, including selenocysteine ligation and various *N*-methylmaleimides, before successfully applying anion-exchange chromatography.

Given that HSP27 requires C137 to slow the entire apoptotic cascade in living cells,^25^ we wondered whether this cysteine could also play an important role in preventing direct caspase-3 activation. First, we recombinantly expressed wild-type HSP27 and directly compared it to HSP27 (C137A) using the same blotting and caspase-3 assays (Figure 3a,b). Interestingly, we found that wild-type HSP27 was a better inhibitor of caspase-3 activation than the corresponding C137A mutant. Therefore, we next set out to synthesize HSP27(gT184) containing the native C137. In our original synthesis of HSP27 (Figure 4), we used an intein fusion to generate a protein thioester corresponding to residues 1–172 of HSP27 still containing C137. We then ligated this protein thioester to O-GlcNAc modified peptides comprising residues 173–205 bearing N-terminal cysteines. After purification of the ligation product by HPLC, we performed a desulfurization reaction that transformed cysteine 173 (C173) to the native alanine but also introduced the C137A mutation. Recently, the Becker and Payne laboratories used similar fragments and phosphorylated peptides with an N-terminal selenocysteine to facilitate the ligation but also allow for selective deselenization and retention of C137.^28^ We decided to attempt this strategy with our modified O-GlcNAc modified peptide. Unfortunately, we were unable to separate the unreacted N-terminal protein thioester (residues 1–172) from the ligation product using a variety of RP-HPLC conditions (example in Figure S1a).

We then decided to take a protecting group approach and chose one developed by the Brik lab, where cysteines can be reversibly reacted with *N*-methyl maleimide reagents that preserve them during the desulfurization reaction,^29^ although the phenacyl protecting-group^30^ has been used to make HSP27 in the past.^31^ As schematized in Figure 4, this method would, on paper, allow us to protect C137, perform the ligation reaction, and then selectively desulfurize C173, giving HSP27(gT184). Accordingly, we reacted the N-terminal HSP27 thioester fragment with *N*-methylmaleimide; however, we were again unable to separate the protected product from the unreacted N-terminal starting material (Figure S1b). We reasoned that even if we were able to push this reaction to completion, it would not resolve our issue from the selenocysteine ligation where we observed coelution of the N-terminal thioester with the full-length HSP27(gT184). Therefore, we next attempted to shift the retention time of the protected N-terminal thioester through the synthesis and application of a variety of modified maleimide-reagents (Figure 3). First, we generated maleimide-containing peptides with either 2, 4, or 6 lysine or 8 arginine residues. Unfortunately, and somewhat surprisingly, we observed only small differences in the HPLC profiles after reaction that could not be resolved from the unreacted starting material or, by extension, the ligation product (Figure S1b). Previously, we found that multiple per-*O*-acetylated O-GlcNAc modifications can result in large changes in RP-HPLC retention times. Therefore, we next prepared a triply O-GlcNAc-modified maleimide peptide; however, this reagent was again unable to shift the N-terminal retention time (Figure S1b).

At this stage, we decided to abandon RP-HPLC despite its place as the gold standard in synthetic protein purification and moved to ion exchange chromatography. We were initially unenthusiastic about this approach, as the pI for the N-terminal fragment and full-length HSP27 only differ by ∼0.5. However, we decided to push forward by first taking the N-terminal thioester protected with the simple *N*-methylmaleimide and reacting it under ligation conditions with the O-GlcNAc modified peptide. This yielded a mixture of full-length protein and N-terminal starting material after RP-HPLC. We subsequently performed the desulfurization and maleimide-deprotection reactions and subjected the reaction mixture to RP-HPLC followed by anion exchange (Figure S2). Gratifyingly, we observed good separation and were able to obtain pure HSP27(gT184) (Figure S3), and the mixed fractions from anion exchange can be resubmitted to the same purification to yield more material.

With wild-type HSP27(gT184) in hand, we performed the direct activation of pro-capase-3 by caspase-9 and visualized the activity using a fluorescent peptide reporter assay (Figure 3c). Similar to the C137A mutant, we found that O-GlcNAc improves the antiapoptotic activity of HSP27. Together, these results suggest that unlike the upstream inhibition of apoptosome formation, C137 is not absolutely required for binding to pro-caspase-3 and blocking its activation by caspase-9, but that it does positivity contribute to this HSP27 activity. Furthermore, they show that, in either case, O-GlcNAc further activates HSP27 in this assay.

## Conclusions

O-GlcNAc and septic responses, including HSP27, is well documented to play important roles in cell survival and the inhibition of apoptosis. For example, the overall levels of both O-GlcNAc and HSP27 are elevated in many cancers and play important roles in tumor survival. However, no direct links between this PTM and protein in the apoptotic cascade had been previously identified. Using protein synthesis, we discovered that O-GlcNAc improves the ability of HSP27 to prevent the activation of the executioner caspase-3 by the upstream caspase-9 *in vitro*. It is unlikely that 100% of HSP27 in a given oligomer would be modified by the oligomer, but our previous results on HSP27 interactions with Aβ(1–42) suggest that substoichiometric O-GlcNAc would be enough to yield better caspase-3 binding. However, we did not directly test that possibility here. Notably, we have previously shown that O-GlcNAc modification of the initiator caspase-8 can directly inhibit its activation, suggesting that the modification may affect multiple nodes of the apoptotic cascade.^32^ Additionally, we show that the redox-active C137 contributes to this inhibition but is not absolutely required, unlike in upstream apoptosis steps in cells. We believe that these results, although currently confined to the test tube, have potentially interesting implications for human disease. In addition to the example of cancer given above, HSP27 is known to be upregulated in neurodegenerative diseases and increasing O-GlcNAc using OGA inhibitors slows the progression of the same diseases in several animal models.^33^ Our previous work showed that the O-GlcNAc modification of HSP27 can function to inhibit the formation of toxic amyloid aggregates that are characteristic of these diseases. The work presented here shows that this same modification may also play a role in preventing apoptosis in the effected neurons, yielding a multifaceted mechanism by which O-GlcNAc and HSP27 may collaborate to protect against progressive neuronal loss.

During our synthesis, we explored the potential for different maleimide reagents to simplify the purification of ligation products and starting material by RP-HPLC. Somewhat surprisingly, and at least in the case of HSP27, we found that these modifications had very little effect on protein retention times and did not enable us to take advantage of RP-purification, the most widely used approach in protein synthesis. However, it is possible that engineering the maleimide protecting groups may be beneficial in the synthesis of other proteins.

## Supporting Information Available

Supplemental figures, experimental procedures, characterization of proteins (PDF)
