# Supplementary material for: HSP27 Inhibitory Activity against Caspase-3 Cleavage and Activation by Caspase-9 Is Enhanced by Chaperone O-GlcNAc Modification in Vitro
Source: ACS Chem Biol. 2023 Jul 14;18(8):1698–704. doi: 10.1021/acschembio.3c00270 (PMC10442853; doi:10.1021/acschembio.3c00270)
Supplement: Supplementary file 1 — cb3c00270_si_001.pdf [file cb3c00270_si_001.pdf]

Supporting Information

HSP27 inhibitory activity against caspase-3 cleavage and activation by caspase-9 is enhanced by chaperone O-GlcNAc modification *in vitro*.

Binyou Wang,[1] Stuart P. Moon,[1] Giuliano Cutolo,[1] Afraah Javed,[1] Benjamin S. Ahn,[1] Andrew H. Ryu,[1] and Matthew R. Pratt[1,2,*]

[1]Departments of Chemistry and [2]Biological Sciences, University of Southern California, Los Angeles, CA 90089, United States

*Corresponding author: Matthew R. Pratt, matthew.pratt@usc.edu

Table of contents:

Figure S1. Unsuccessful methods to synthesize HSP27(gT184) (wt). **Page S2**
Figure S2. Purification of HSP27(gT184) (wt) by anion exchange. **Page S3**
Figure S3. Characterization of HSP27(gT184) proteins. **Page S3**

Materials and Methods **Page S4**

References **Page S6**

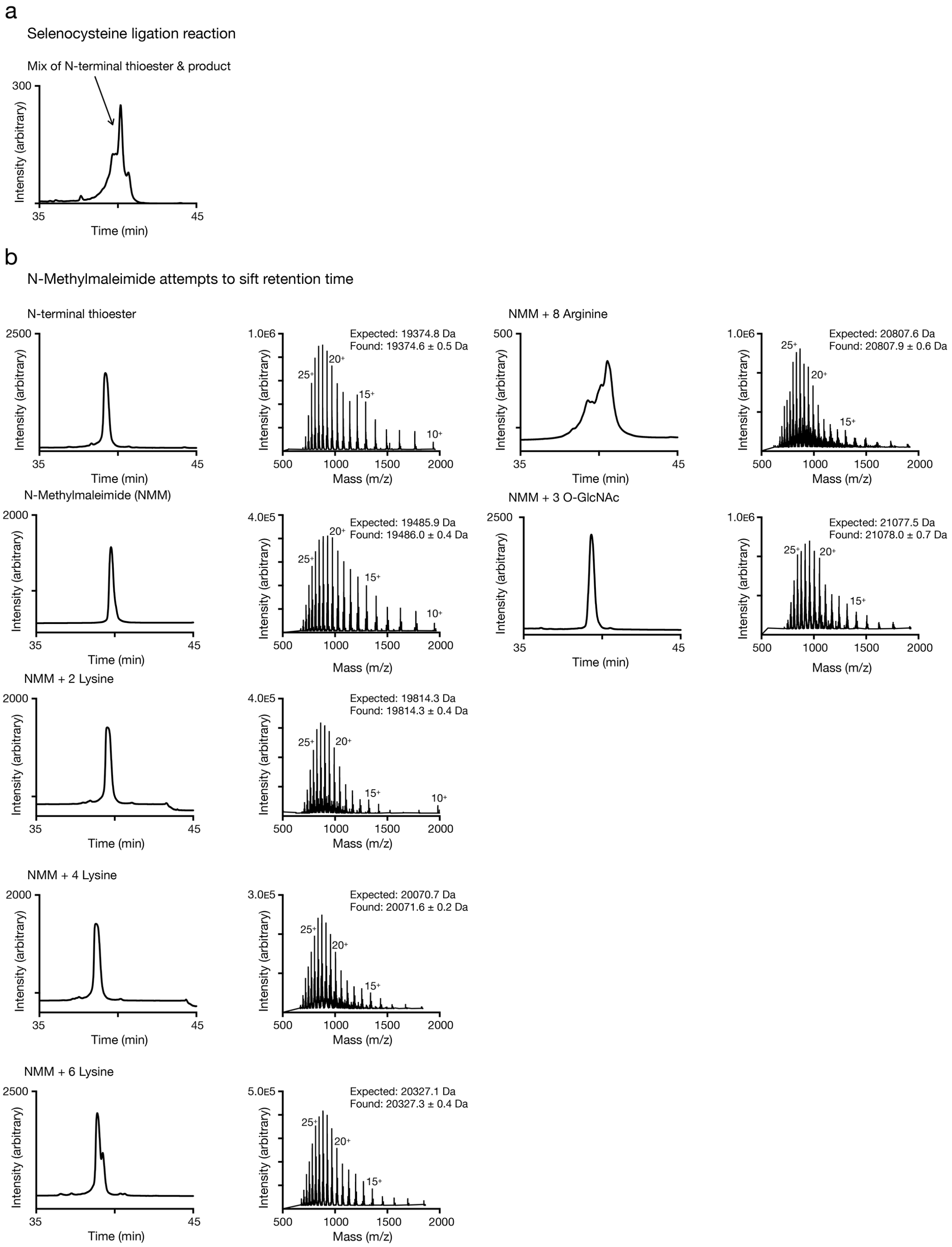

Figure S1. Unsuccessful methods to synthesize HSP27(gT184) (wt). a) RP-HPLC trace of the selenocysteine ligation showing an inseparable mixture of the ligation product and N-terminal thioester. b) Various N-methylmaleimides do not notably shift the retention time of the N-terminal thioester. RP-HPLC traces and ESI-MS characterization of corresponding *N*-methylmaleimide reactions.

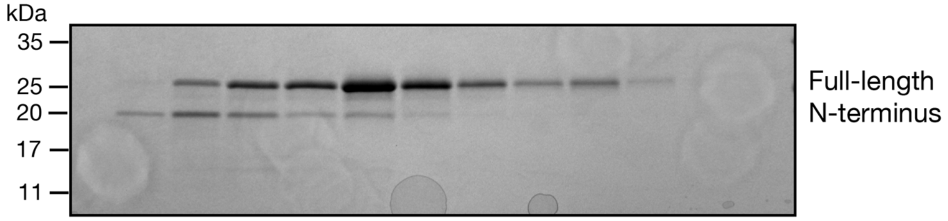

Figure S2. Purification of HSP27(gT184) (wt) by anion exchange. Fractions from anion-exchange chromatography were analyzed by SDS-PAGE and Coomassie staining showing separation between the N-terminal fragment and final HSP27(gT184) (wt) product.

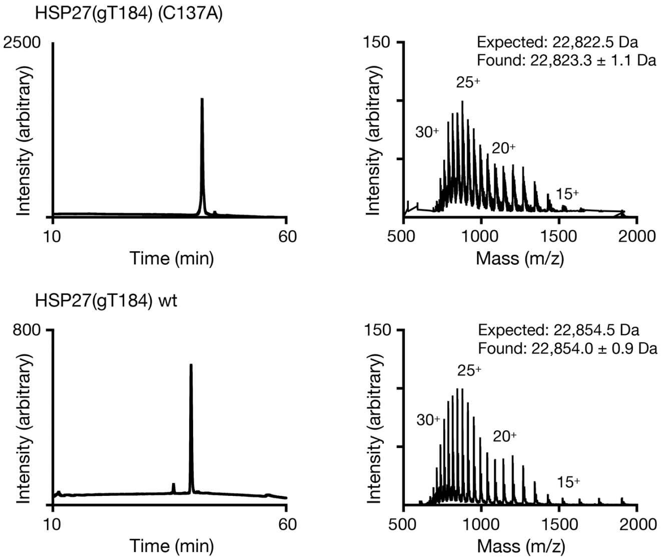

Figure S3. Characterization of HSP27(gT184) proteins. RP-HPLC traces and ESI-MS characterization of HSP27(gT184) (C137A) and HSP27(gT184) (wt).

General

All materials were purchased from commercial vendors and used without prior purification. Aqueous solutions were prepared using ultra-pure water. Bacteria broth was prepared and sterilized following vendor instructions, and cultures were handled and manipulated aseptically. Protein concentrations were determined by BCA assay (Thermo). Reverse-phase high performance liquid chromatography (RP-HPLC) was performed on an Agilent 1200 Series HPLC with a diode array detector fitted with analytical or semipreparative C4 or C18 Phenomenex columns (Buffer A: 0.1% trifluoroacetic acid (TFA), Buffer B: 90% acetonitrile, 10% water, 0.1% TFA). ESI-MS was performed on an Aglient 6545 qTOF mass spectrometer with LC front end.

Generation of plasmids

The generation of plasmids used in this work has been described previously.{Balana.2021gmi} Site-directed mutagenesis was performed according to manufacturers instructions using Agilent QuikChange Lightning kits to install the C137A point mutation.

Preparation of HSP27 2-172 thioester

Plasmid DNA encoding HSP27(2-172)-AvaE-6xHis fusion was transformed into BL21(DE3) (EMD Millipore) cells via heat shock and selected with ampicillin plates (100 μg ml^{-1}). A starter culture (Luria broth, 100 μg ml^{-1} ampicillin) was grown overnight at 37 °C overnight from a single colony. Three liters of Terrific broth were then inoculated with the overnight culture and grown to an OD 600 of 0.6-0.8 at 37 °C while being shaken at 225 rpm. Protein expression was induced with the addition of IPTG at a final concentration of 1 mM and proceeded for 6 h at 37 °C with shaking at 225 rpm. Cells were harvested at 6,000 x g, and the resulting pellets were resuspended in lysis buffer (20 mM NaH_2PO_4, 250 mM NaCl, 1 mM TCEP, 5 mM imidazole, and 2 mM PMSF, pH 7). GuHCl was added to give a concentration of 6M before performing tip sonication on ice (75% amplitude, 30 s on, 30 s off, total of 5 min), and the lysate was clarified by centrifugation (20,000 x g for 30 min at 4 °C). Supernatants were loaded onto Co-NTA agarose beads (GoldBio) and washed extensively (20 mM NaH_2PO_4, 300 mM NaCl, 20 mM imidazole, 4M urea, pH 7). Protein was then eluted (20 mM NaH_2PO_4, 300 mM NaCl, 1 mM TCEP, 250 mM imidazole, 4M urea, pH 7). Excess imidazole was then removed by buffer exchange with 4M urea in 1X DPBS using 10k centrifugal filters (Amicon Ultra, EMD Millipore). The protein thioester was generated through transthioesterification by the addition of sodium mercaptoethanesulfonate (MESNa) at a final concentration of 250 mM at pH 7 followed by incubation at room temperature for 2 d. The final product was purified by semipreparative RP-HPLC and pure proteins were characterized by analytical RP-HPLC and ESI-MS. Purified proteins were lyophilized prior to storage. Typical yield was 1.5 mg L^{-1} of culture.

Peptide synthesis of HSP27 173-205 (gT184)

Standard manual Fmoc-based solid phase peptide synthesis (SPPS) was performed in DMF using Fmoc-Lys (Trt)-pre-loaded Wang resin (P3Bio). Commercially available N-Fmoc and side-chain-protected amino acids (5 equiv, P3Bio) were activated for 5 min with HBTU (4.5 eq, Novabiochem) and N,N-diisopropylethylamine (DIEA) (10 eq) and coupled to the resin for 1h with constant agitation. Between each coupling step, the resin was washed and the N-terminal Fmoc group was removed with 20% v/v piperidine for 15 mins in total. *N*-terminally and *O*-acetyl protected O-GlcNAcylated threonine was prepared as an *O*-pentafluorophenyl (Pfp)-activated ester as previously described.[1] Two equivalents of Pfp-activated O-GlcNAcylated threonine were used during coupling without HBTU or DIEA in an extended overnight incubation. Peptides were cleaved from the resin with the addition of standard cleavage cocktail (95:2.5:2.5 TFA/H2O/triisopropylsilane) for 4 h at room temperature. The peptides were precipitated in pre-cooled ether and incubated overnight (-80 °C). The peptides were collected by centrifugation (15 min, 6,000 x g, 4 °C), and resuspended in 50:50 acetonitrile:H_2O, flash frozen, and lyophilized. The crude peptide mixture was purified by RP-HPLC. Typical yield of peptide was 4%.

Synthesis of *N*-terminal maleimide peptides

Peptides functionalized with maleimide at their N-termini were generated using SPPS as above with a final coupling of 4-maleimidobutyric acid using standard HBTU/DIEA conditions prior to cleavage. Peptides were purified via RP-HPLC and characterized by mass spectrometry. **2K-NMM** expected: 439.51 Da, observed: 439.25 Da. **4K-NMM** expected: 695.86 Da, observed: 695.44 Da. **6K-NMM** expected: 952.2 Da, observed:

951.6 Da. **8R-NMM** expected: 1432.7 Da, observed: 1432.9 Da. **3gT-NMM** expected: 1702.6 Da, observed: 1701.6 Da. Typical peptide yields ranged from 10 to 20%.

Maleimide protection of HSP27 2-172 thioester and ligation with HSP27 C-term 173-205 (gT184)
HSP27 2-172 protein thioester was resuspended at a 2 mM concentration in 6 M GuHCl, 250 mM phosphate, pH 7 and cooled to -18 °C. Three equivalents of methylmaleimide and 1 equivalent of TCEP were added, and the product was detected by RP-HPLC and LC/MS after 30 mins incubation at -18 °C with agitation. The protected HSP27 thioester was then purified by RP-HPLC. The protected HSP27 thioester was concentrated to 5 mM in ligation buffer (6 M guanidine, 250 mM phosphate, 25 mM TCEP, 25 mM MPAA pH 7) before the addition of 1.5 equivalents of HSP27 C-terminal peptide. Following overnight ligation, the N-term/product mixture was purified with RP-HPLC.

Desulfurization, maleimide deprotection, and acetate deprotection of HSP27
The lyophilized mixture of N-term and ligation product was resuspended at a 2 mM concentration in degassed 6 M guanidine, 250 mM phosphate buffer. VA-044 (2,2'-Azobis[2-(2-imidazolin-2-yl)propane]dihydrochloride, 40 mM), TCEP (175 mM), and glutathione (120 mM) were added to initiate desulfurization. After incubation at 37 °C for 4 h, fully desulfurized HSP27 was detected by ESI-MS. The N-term/ligation product-mixture was purified by RP-HPLC and then dissolved at a 1.5 mM concentration in deprotection buffer (6 M guanidine, 200 mM phosphate, 20 eq $PdCl_2$, 100 eq $MgCl_2$, pH 7) to remove the maleimide protecting group. After 2 h incubation at 37 °C, the reaction was quenched with 40 equivalents of DTT. To remove the sugar's O-acetyl protecting groups, the reaction mixture was adjusted to 5% v/v hydrazine monohydrate and incubated for 1 h at room temperature. The reaction was quenched with 5% v/v acetic acid, and the mixture was reduced by the addition of solid TCEP (5 mg) before RP-HPLC purification and lyophilization.

Selenocysteine ligation
The *N*-terminal selenocysteine peptide was generated using the SPPS conditions described above using Boc-Sec(Mob)-OH prepared in-house as detailed previously.[2] Dithiobis(5-nitropyridine) (1.3 eq) was added to the cleavage solution cocktail following synthesis. Recombinant HSP27 thioester and synthetic modified C-term were resuspended in a 2:1 molar ratio in degassed selenocysteine ligation buffer (6 M GuHCl, 200 mM phosphate, 100 mM ascorbic acid, 50 mM TCEP, 250 mM MPAA, pH 7) to a peptide concentration of 4 mM. Following overnight ligation, the reaction was monitored via HPLC and ESI-MS but formed the mixture of products shown in Figure 3.

Anion exchange chromatography
Either manual or FPLC-assisted (Amersham Pharmacia Biotech ÄKTA FPLC (UPC-900, P-920)) anion exchange was used to separate the *N*-terminal fragment from the ligation product. The protein mixture was suspended in FPLC Buffer A (4 M urea, 20 mM Bis-Tris, pH 7) and loaded onto a HiTrap Q HP anion exchange column (Cytiva Life Sciences). The column was washed with FPLC Buffer A before slowly increasing NaCl concentration with FPLC Buffer B (4 M urea, 20 mM Bis-Tris, 500 mM NaCl, pH 7) to elute each protein species. The elutions were analyzed via SDS-PAGE and Coomassie staining, and pure fractions were pooled and concentrated using centrifugal filters. Purified products were refolded via overnight dialysis against 1X DPBS and concentrated as required. Overall protein yield was ~3% based on the N-terminal thioester.

Western blot analysis of caspase-3 cleavage
Recombinant procaspase-3 (Enzo Life Sciences, 100 ng) was incubated for 30 min on ice with HSP27 proteins in of 5 µL assay buffer (20 mM Tris, 10 mM NaCl, 1mM EDTA, 1mM DTT, pH 7.4). Various amounts of active caspase-9 (0.0025U-0.02U) were added to 5 µL cleavage buffer (50 mM HEPES, 50mM NaCl, 10% Sucrose, 5mM DTT, 1mM PMSF, pH 7.4) to initiate the cleavage before incubation at 37 °C. Processing of caspase-3 was determined by immunoblotting. SDS-PAGE was performed using precast 4-20% polyacrylamide Tris-glycine gels (Invitrogen), which were transferred to PVDF membranes (BioRad). The blots were then blocked using Prometheus OneBlock (Genesee) for an hour at room temperature. An anti-caspase-3 antibody (9662, Cell Signaling Technologies, 1:1000 TBST) was then added overnight at 4 °C, followed by anti-rabbit secondary (711-035-152, Jackson Immunoresearch) for 1h at room temperature. for Western blotting (WB) analysis. Signal was generated using ECL substrate (BioRad), imaged on a ChemiDoc

MP Imaging System (BioRad) and quantified using volumetric densitometry using ImageLab software (6.1.0.07, BioRad).

Caspase-3 DEVD-AFC activity assay

Recombinant procaspase-3 (Enzo Life Sciences, 100 ng) was mixed for 30 min on ice with HSP27 proteins in 10 µL assay buffer (20 mM Tris, 10 mM NaCl, 1mM EDTA, 1mM DTT, pH 7.4) to promote the formation of the HSP27/Caspase-3 complex. The protein complex was then combined with AFC-DEVD peptide substrate (10 µL of a 20 mM stock) in 100 µL cleavage buffer (50 mM HEPES, 50mM NaCl, 10% Sucrose, 5mM DTT, 1mM PMSF, pH 7.4). Active caspase 9 (0.015U) (Enzo Life Sciences) was then added to promote the cleavage of procaspase-3. The reaction solution was transferred to wells of a 96-well plate for fluorescence measurement (λ_{Ex} = 400 nm, λ_{Em} = 505 nm) on an Agilent BioTek Citation 5 plate reader. The fluorescence intensities were read every 5 min for 7 h with continuous shaking at 37 °C.

References

(1) Leon, C. A. D.; Lang, G.; Saavedra, M. I.; Pratt, M. R. Simple and Efficient Preparation of O- and S-GlcNAcylated Amino Acids through InBr 3 -Catalyzed Synthesis of β- N -Acetylglycosides from Commercially Available Reagents. *Org Lett* **2018**, *20* (16), 5032–5035.

(2) Shimodaira, S.; Iwaoka, M. Improved Synthetic Routes to the Selenocysteine Derivatives Useful for Boc-Based Peptide Synthesis with Benzylic Protection on the Selenium Atom. *Arkivoc* **2016**, *2017* (2), 260–271.
